# Stepping Stones to Sustainability Within Cancer Clinical Trials in Ireland

**DOI:** 10.3390/curroncol32080446

**Published:** 2025-08-08

**Authors:** Angela Clayton-Lea, Calvin R. Flynn, Claire Hopkins, Seamus O’Reilly

**Affiliations:** 1 Cancer Trials Ireland, RCSI House, D02 H903 Dublin, Ireland; angela.claytonlea@cancertrials.ie (A.C.-L.); claire.hopkins@cancertrials.ie (C.H.); 2 Medical Oncology, Cork University Hospital, Wilton, T12 DFK4 Cork, Ireland; calvinrflynn@gmail.com

**Keywords:** cancer clinical trials, sustainability, carbon footprint, healthcare emissions, clinical research

## Abstract

Cancer clinical trials contribute significantly to healthcare-related greenhouse gas emissions, highlighting the need to address sustainability in this area as the climate crisis intensifies. This study provides the first national assessment of sustainability awareness, attitudes, and practices within the Irish cancer clinical trials community. A 21-item cross-sectional survey was distributed to 613 cancer research professionals affiliated with Cancer Trials Ireland, including clinicians, research nurses, trial coordinators, patient advocates and industry staff, yielding a 20.6% response rate. Survey items assessed awareness of sustainability tools, perceived carbon contributors, training received, confidence in implementing green practices, and perceived barriers and enablers to sustainability. Awareness of existing carbon footprint tools was low, with only 21% familiar with the Sustainable Clinical Trials Group guidelines and fewer than 6% aware of the National Institute for Health and Care Research calculator. Despite limited training and low confidence in implementing carbon-reductive measures, 86% of respondents expressed willingness to engage with sustainability initiatives. Trial-related travel, sample kit waste, and trial set-up were perceived as the highest contributors to emissions, though perceptions did not always align with published data. Key barriers included lack of education, institutional support, and regulatory clarity, while financial incentives and training were identified as enablers. Coordinated, system-wide interventions are needed to embed sustainability into cancer clinical trial design, governance, and funding processes.

## Simple Summary

Cancer clinical trials are essential for advancing treatment but also contribute to carbon emissions through travel, energy use, and waste. This study surveyed cancer research professionals across Ireland to explore their awareness and attitudes toward sustainability. Most had not received formal training but were enthusiastic about reducing emissions through digital tools, less travel, and waste reduction. Respondents called for clearer national guidance and institutional support. These findings suggest strong interest in greener clinical trials and highlight Ireland’s opportunity to lead in sustainable cancer research.

## 1. Introduction

Climate change represents a defining challenge of our era, with profound implications for human health and the healthcare sector. The Intergovernmental Panel on Climate Change (IPCC), in its 2023 Sixth Assessment Synthesis Report, warned of “a rapidly closing window of opportunity to secure a livable and sustainable future for all” [1]. The World Health Organization (WHO) has echoed these concerns, describing climate change as a fundamental threat to global health, capable of reversing decades of progress in development and widening health inequities [2].

Healthcare systems are both vulnerable to climate change and significant contributors to it. The global healthcare sector accounts for approximately 4.6% of net greenhouse gas (GHG) emissions, more than double that of the aviation industry [3,4]. These emissions arise not only from direct activities, such as fuel combustion and facility operations, but also from indirect sources across the energy supply chain and procurement systems. These are commonly categorized as Scope 1 (direct emissions), Scope 2 (indirect emissions from purchased electricity), and Scope 3 (all other indirect emissions, including those from supply chains and service delivery) [5]. In high-income countries, healthcare contributes 3–8.5% of national emissions, including 7.6% in the United States (US) and 5.4% in the United Kingdom (UK) [3].

Cancer care, encompassing surgery, systemic anti-cancer therapy, radiation therapy, and clinical research, has a particularly high environmental burden. Overtreatment, waste from single-use plastics and cytotoxics, and energy-intensive treatment modalities contribute significantly to healthcare’s carbon footprint [6,7,8]. Clinical trials, a cornerstone of oncology innovation, are increasingly recognized as carbon-intensive due to extensive travel, resource consumption, and expanding data storage needs. Notably, trials often collect large volumes of redundant data, with one analysis finding that just 5% of collected data informs the primary endpoint, and only 20% is used in the final publication [9], highlighting opportunities for carbon reduction through streamlined trial design. One estimate places the carbon footprint of global clinical research at 100 million tons of carbon dioxide equivalent (CO_2_e) annually [10], with cancer trials accounting for over 100,000 active studies worldwide.

Despite this, few tools exist to quantify or mitigate the carbon footprint of clinical trials. While some efforts, such as the Sustainable Clinical Trials Group (SCTG) guidelines [11] and more recent initiatives by the Low Carbon Clinical Trials Working Group [12], have proposed frameworks and calculators, widespread adoption remains limited. Integration of sustainability into trial design is hampered by infrastructure gaps, limited institutional awareness, and entrenched design practices [13]. Previous international studies have documented low levels of climate literacy, limited engagement with emissions reduction strategies, and variable climate advocacy among health professionals, despite growing interest in sustainable healthcare practices [14,15,16]. A recent survey by Hoffmann et al. [13] revealed that over 60% of academic and industry stakeholders were unaware of sustainability measures in trial design.

Ireland’s 2025 National Climate Change Risk Assessment recognizes the healthcare sector as both vulnerable to and responsible for climate risk, calling for cross-sectoral action, including research and innovation [17]. Ireland is uniquely positioned to lead in sustainable cancer research within Europe. As the European Union (EU)’s largest net pharmaceutical exporter [18] and host to nine of the world’s ten largest pharmaceutical companies [19], the country has a well-established research infrastructure. Cancer Trials Ireland (CTI), the national coordinating body for oncology clinical trials, is well-positioned to take a leadership role in this domain. CTI oversees research across seven Health Research Board (HRB)-funded university-hospital clusters and also has strong industry collaboration, with 47% of CTI trials being industry-sponsored. However, a study by Myo Oo et al. has previously found that climate advocacy among cancer clinical trial organizations remains limited [20], highlighting the need for greater institutional engagement in sustainability efforts.

This study explores the intersection of cancer clinical trials and environmental sustainability in Ireland. Its primary aim is to assesses awareness, attitudes, and current practices related to the carbon footprint of trials. The secondary aim is to identify perceived barriers and enablers to sustainable practices, and to explore opportunities for integrating sustainability into trial design and conduct.

## 2. Materials and Methods

### 2.1. Study Design

We conducted a cross-sectional survey incorporating both quantitative and qualitative elements to explore sustainability practices in cancer clinical trials in Ireland. A pragmatic approach was adopted to generate practical, real-world insights rather than test a specific hypothesis. Data analysis was guided by abductive reasoning, allowing themes to emerge inductively from participant responses while remaining grounded in existing frameworks and evidence.

### 2.2. Survey Design

A structured, 21-item questionnaire was developed to explore: (1) knowledge of the carbon footprint associated with cancer clinical trials, (2) awareness of sustainability-enhancing innovations, and (3) perceived barriers and incentives to implementing such measures. Questions were informed by existing literature and tools, including the SCTG guidelines [11], the National Institute for Health and Care Research (NIHR) sustainability assessment tool [21], ‘My Green Lab’ (MGL) Certification [22], and publications by Hoffmann et al. and Griffiths et al. [12,13]. The questionnaire included both closed and open-ended formats, with “don’t know” options where appropriate. The questionnaire was piloted with a consultant medical oncologist, resulting in minor refinements for clarity and scope. A summary of the survey domains is provided in Table 1.

### 2.3. Participants and Recruitment

Participants were cancer research professionals affiliated with CTI, including those from clinical, academic, and industry backgrounds. The survey was distributed via email to 613 individuals using CTI’s membership database. Respondents were purposively sampled from the CTI database to ensure representation across research roles, cancer subspecialties, and geographical locations.

### 2.4. Data Collection Process

Data collection was conducted through SurveyMonkey over a three-week period beginning 3 April 2024. Participation was voluntary and anonymous. Informed consent was obtained on the survey landing page, which detailed the study objectives, data protection measures, and rights to withdraw. No personal identifiers or IP addresses were collected. Data were securely stored and scheduled for deletion six months post-analysis.

### 2.5. Ethical Considerations

The study was conducted in accordance with the Declaration of Helsinki. As this was a non-interventional study involving anonymized data, formal ethical approval was not required. Ethical considerations were informed by principlist and care-based frameworks, ensuring confidentiality, data security, and respect for participants.

### 2.6. Data Analysis

Quantitative data were analyzed using descriptive statistics to summarize categorical responses. Open-text responses were analyzed using thematic analysis as described by Braun and Clarke [23].

## 3. Results

### 3.1. Demographics

A total of 126 individuals responded to the survey out of 613 invited members and stakeholders of CTI, yielding a response rate of 20.6%. Respondents represented a wide range of professional roles across the clinical, academic, and industry sectors (see Table 2). The majority were hospital-based clinicians, research nurses, or clinical trial coordinators, with additional input from principal investigators, trial managers, and stakeholders from sponsor organizations and contract research organizations (CROs). Representation spanned all four provinces of Ireland, with the largest proportion based in Leinster (63%).

### 3.2. Awareness and Knowledge of Carbon Footprint Tools

Awareness of carbon footprint assessment tools specific to clinical trials was low among respondents. Of the 71 individuals who answered this question, 68% reported no awareness of established approaches such as the SCTG guidelines, the NIHR carbon footprint calculator, or MGL certification. Only 21% were aware of the SCTG guidelines, 20% were aware of MGL certification, and fewer than 6% had heard of the NIHR calculator. A small minority (4%) indicated involvement in other approaches, including paperless trials and use of electronic site files.

In Questions 7 and 8, respondents were asked about the extent to which they believed carbon footprint is considered during the design phase of industry-sponsored versus academic clinical trials. Among the 72 respondents to each question, 49% believed sustainability was rarely considered in industry-sponsored trials, compared to 43% for academic trials. A further 29% thought it was occasionally considered in academic trials, while only 15% believed the same for industry-led studies. Very few felt it was considered most of the time; just 2.8% for industry and 4.2% for academic trials. Notably, a large proportion selected “don’t know,” with 33% unsure about industry trials compared to 24% for academic trials. Most responses were based on opinion rather than direct knowledge, with 36% indicating this for industry trials and 39% for academic. Additionally, more respondents had direct experience with academic trials (14%) compared to industry-sponsored (4%). These findings highlight a widespread lack of clarity and direct exposure to sustainability practices during trial design, particularly in the industry sector. Free-text comments reinforced this, with several respondents noting that environmental sustainability is rarely discussed or incentivized during protocol development stages.

### 3.3. Perceived Contributors to Trial Emissions

In Question 9, respondents were asked to rank ten trial-related activities in order of their perceived impact on a trial’s carbon footprint, with 1 indicating the highest impact and 10 the lowest. A total of 70 respondents completed this ranking task. The activities were selected based on literature, including Griffiths et al. [12], to align with categories used in the NIHR carbon footprint calculator.

Trial-specific meetings and travel were perceived as the most significant contributors, receiving the highest weighted average score (7.31), and were ranked among the top four by 48 respondents. This was closely followed by trial supplies and equipment (score: 7.13; ranked in top four by 46 respondents). Other high-impact categories included collection and shipment of samples (6.57), emissions from Clinical Trial Research Units (CTRUs) (6.52), and trial set-up activities (6.41).

In contrast, trial close-out was consistently viewed as the least impactful, with 54% of respondents ranking it lowest. Additional lower-impact activities included patient assessments (ranked 8th by 29%) and laboratory-related activities (ranked 9th by 24%).

These findings suggest that travel, both for staff and trial activities, is viewed as the leading source of emissions, consistent with existing research. Notably, several respondents commented on their uncertainty in assessing the relative carbon impact of these activities, citing a lack of knowledge or tools. This highlights a broader issue of limited carbon literacy within the cancer clinical trials workforce.

### 3.4. Training, Confidence, and Willingness to Implement Sustainable Practices

In Question 10, respondents were asked whether they had received any education or training in their workplace on measuring or reducing the carbon footprint of clinical trials. Of the 71 individuals who answered this question, 97% reported that they had not received any formal training, highlighting a significant gap in structured education on environmental sustainability within the cancer trials sector. Only two respondents (3%) indicated they had received such training, one was a translational scientist currently working as a Principal Investigator, while the other did not specify their professional role.

Question 11 explored perceived competence in advising on or implementing carbon-reductive measures within trials. Of the 71 respondents, only 2.8% (*n* = 2) felt very confident in their ability to do so. A further 17% believed they could contribute with some assistance, whereas the vast majority (82%) indicated that they would require significant assistance or guidance to take meaningful action in this area.

Despite this lack of confidence and training, responses to Question 12 revealed a strong underlying enthusiasm. When asked about their willingness to engage in sustainability initiatives, 41% of respondents stated they would be willing to participate in advising on or implementing sustainability measures. An additional 45% expressed interest but noted that their current role would not allow for such involvement.

### 3.5. Perceptions of Innovative Measures to Reduce Carbon Footprint

To address the second objective, assessing awareness of the potential impact of innovative measures to reduce the carbon footprint of cancer clinical trials, respondents were asked in Question 13 to select five measures they believed would have the greatest impact. Of the 71 who responded, only 10% indicated they lacked sufficient knowledge to make selections, suggesting a generally high level of engagement with the topic.

The most commonly selected innovation was reducing sample kit waste (59%), followed by full implementation of electronic patient records (55%), and virtual assessments for patient follow-up (54%). Other frequently selected measures included streamlining ethics and regulatory approvals and reducing patient site visits. Conversely, membership in Green Lab initiatives and establishment of a national biobank were selected least often, possibly reflecting lower familiarity or perceived feasibility among respondents.

In Question 14, respondents were invited to share additional practical suggestions for reducing a trial’s carbon footprint. Eighteen responses were submitted and analyzed thematically using Braun and Clarke’s method. Fifteen themes emerged, eight of which were unrelated to the list in Question 13, demonstrating the breadth of ideas among stakeholders. Locally focused suggestions included resource-sharing across trial units, evaluating the energy efficiency of storage practices, and consolidating site monitoring to a single Clinical Research Associate. Externally oriented proposals included linking sustainability performance to trial accreditation or funding, engaging regulators in environmental accountability, and minimizing the use of shipping materials (e.g., gel packs, dry ice) in industry-sponsored trials.

Question 15 invited respondents to describe any observed or known impacts resulting from the implementation of such innovations. Six participants provided input. While there was no clear thematic convergence, the examples were diverse and informative. Reported initiatives included raising freezer temperatures from −80 °C to −70 °C, using electronic investigator site files, collecting adverse event data on iPads, emailing patient information leaflets, and conducting fully paperless trials with electronic databases. One respondent referenced the development of a green charter and trial carbon calculator.

### 3.6. Barriers and Incentives to Sustainable Practice in Cancer Clinical Trials

To address the third research objective, identifying perceived barriers and incentives to adopting sustainable practices, respondents were asked a series of structured and open-ended questions.

**Figure 1:**
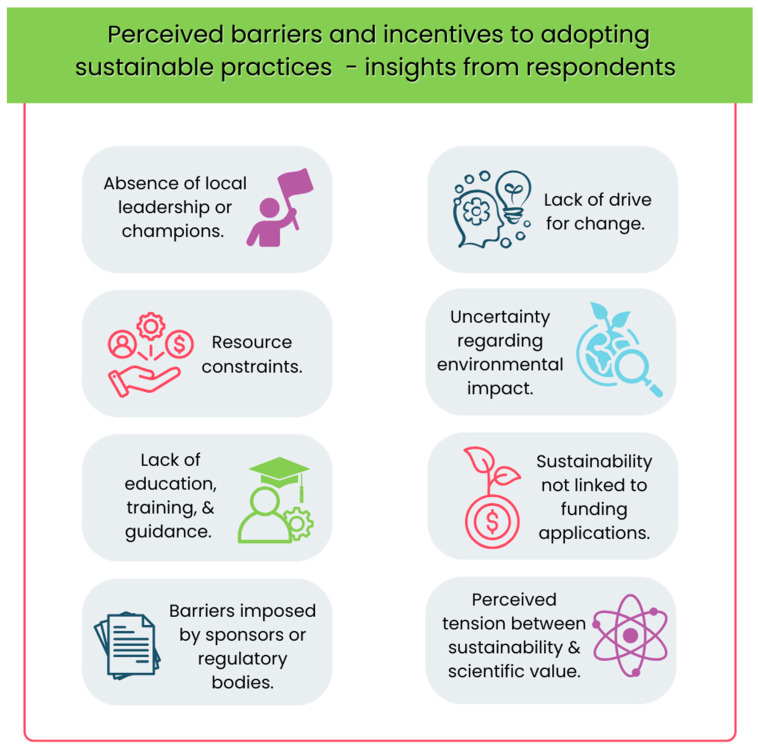
Perceived barriers and incentives to adopting sustainable practices—insights from respondents.

In Question 16, 64 respondents selected their three main perceived barriers from a list of nine options (see Figure 1). The most commonly cited barrier was the lack of education, training, and guidance (66%), followed by resource constraints (39%) and lack of drive for change (33%). Other barriers included perceived tension between sustainability and scientific value, uncertainty regarding environmental impact, and absence of local leadership or champions. Additional comments revealed that some respondents viewed the primary barriers as external to their organization, particularly those imposed by sponsors or regulatory bodies. For example, one participant noted: “Many people just don’t think this is an important issue” (16:CM08), while another criticized the redundancy of reprinting lengthy patient information leaflets for reconsent (16:CM05).

In Question 17, 23 participants described personal experiences with these barriers. Thematic analysis revealed recurring issues such as time constraints, resistance to change, staff shortages, and the perception that sustainability is not prioritized institutionally. Respondents also flagged rigid paper-based documentation, inflexible sample kit protocols, and ongoing requirements for wet signatures as impediments to greener practice. One response encapsulated the existential challenge of climate action within the clinical research community: “I think people feel overwhelmed by climate change… our work is about our health but our focus is on treating illness” (17:CM07). Others identified regulators as crucial enablers of change, with one respondent remarking: “I’ve seen how simple changes from regulators can enable much more efficient processes. Hence I believe regulators are key” (17:CM19). A call for CTI to lead institutional change also emerged (17:CM03).

In Question 18, 15 participants offered suggestions for overcoming these barriers. Frequently cited solutions included protected time, training, guidance, and additional resources. One respondent proposed enabling multi-study programming on a single machine as an example of pragmatic change for industry trials (18:CM06), while another recommended: “National guidance to allow/promote direct to electronic database data entry… needs advocacy” (18:CM02). Incremental improvement was also encouraged: “Don’t try to change everything at once” (18:CM12).

In Question 19, 65 respondents selected three key facilitators to incentivize sustainability. Financial support to introduce electronic trial master files ranked highest (52%), followed by guidance and training (49%), and mandatory inclusion of sustainability measures in grant applications (45%). The least favored facilitator was patient/public awareness (12%). Free-text suggestions reinforced the importance of regulatory flexibility, inclusion of sustainability costs in budgets, and linking funding to sustainability compliance, with one respondent stating: “I think you have to make funding contingent on sustainability—that’s the only way you will get the buy-in needed” (19:CM01).

Questions 20 and 21 gathered open feedback. Ten respondents commented on how to accelerate sustainable practice, highlighting 12 themes. Key suggestions included making sustainability mandatory in funding applications, introducing trial set-up carbon checklists, and offering practical guidance for time-poor clinicians (20:CM05). One respondent suggested sustainability become a mandatory module for all CTI members, while another recommended a ‘green trial’ social media campaign to raise awareness (21:CM08).

The need for education was a consistent theme: “Education is key to driving change” (21:CM05). Others stressed the role of IT infrastructure and predicted future EU legislation would likely mandate action: “We need to educate trial leads as to just how important this will be within 5 years” (21:CM07).

Overall, while barriers such as limited training, competing priorities, and perceived regulatory hurdles persist, respondents identified clear facilitators to support change. These include national guidance, continuous professional development (CPD)-accredited training, toolkits, and the appointment of green champions. The findings suggest that the Irish cancer clinical trials community is open to sustainable innovation, but meaningful progress will require system-wide alignment, structural incentives, and cultural transformation.

## 4. Discussion

The study drew participation from a multidisciplinary cohort of cancer research professionals in Ireland, with consultant medical oncologists, consultant radiation oncologists, consultant hematologists, and translational scientists collectively accounting for 63% of respondents. Surgeons constituted only 4% of the sample, which is noteworthy given the substantial role of surgery in cancer care and its associated environmental impact. Over half of participants (*n* = 57) currently hold or have previously held senior roles in clinical trial research, placing them in positions of influence regarding trial design, process innovation, and leadership. All major clinical trial roles were represented, including members of ethics boards, patient advocacy groups, and individuals from academia, private healthcare, and cancer charities, reflecting a comprehensive cross-section of the cancer trials ecosystem.

The findings revealed a significant gap in awareness and training regarding sustainable clinical trial practices. Although guidance and tools such as Hoffmann et al.’s carbon reduction checklist [13] and the SCTG Guidelines [11] exist, the majority of respondents were unfamiliar with these resources. Only 21% were aware of the SCTG Guidelines and 20% of the MGL Certification program [22]. Awareness of the more recent NIHR guidelines [21] was particularly low (<6%). This limited engagement with existing tools underscores a broader issue; while clinicians and research professionals are increasingly recognized as key agents in climate action [24], many lack the institutional support and training needed to fulfill this role within the context of clinical trials. This aligns with international evidence highlighting a lack of integration of climate change into health curricula and training programmes, as well as broader gaps in climate literacy and engagement among health professionals [25,26,27,28,29,30]. Dablander et al. found that climate scientists report significantly higher levels of climate-related advocacy and personal behavior change compared to non-climate researchers [31], suggesting that domain-specific knowledge may strongly influence sustainability engagement. Recent evidence also suggests that individuals who attribute extreme weather events to climate change are significantly more likely to support climate policy initiatives, regardless of actual exposure [32].

This lack of familiarity extended to competence in using carbon footprint calculators, with most respondents expressing limited knowledge or experience. These findings mirror previous research by Carlberg and Jansson [33], Huang et al. [34], Elia et al. [35], and Rosa et al. [36], which similarly identified the lack of education and training as a key barrier to adoption of sustainable practices. Encouragingly, despite these deficits, the majority of respondents expressed willingness to engage with sustainability initiatives, suggesting a readiness to act if appropriately supported. To address this gap, there is a strong rationale for a coordinated education and awareness campaign, potentially through a CPD-accredited training program, highlighting available tools, including the forthcoming industry-wide Low Carbon Clinical Trials (iLCCT) online platform [37]. CTI, as the national coordinating body, is well-positioned to lead the development of sustainability-focused training modules for clinical trial staff. Similar findings were reported by O’Reilly et al. among Breast International Group members, where respondents expressed strong support for sustainability integration but cited practical and institutional barriers [38].

Most respondents indicated that the carbon footprint of clinical trials is rarely considered during the design phase, a finding that aligns with the work of Hoffmann et al. [13]. While some respondents suggested that such considerations may be more common in academic-led studies, a significant proportion expressed uncertainty. Hoffmann et al. also found that GHG emissions are generally not assessed during Institutional Review Board (IRB) submissions, suggesting that even in academic contexts, environmental impact is not routinely incorporated into trial design. These findings collectively highlight a broader gap in the integration of sustainability principles into the early planning stages of cancer clinical trials.

When evaluating knowledge of emissions from specific trial-related activities, there were notable discrepancies between respondent perceptions and empirical data from Griffiths et al. [12] and Mackillop et al. [39]. For instance, respondents ranked trial set-up and sample shipment as major contributors to emissions, whereas Griffiths et al.’s empirical calculations placed these activities lower [12]. While some variation in rankings may reflect trial-specific characteristics (e.g., internal flights or multiple radiotherapy visits), the findings underscore a need for greater clarity and standardization in calculating and communicating the environmental impacts of different trial activities.

The majority of respondents viewed the reduction in sample kit waste as the most impactful innovative strategy, aligning with previous calls by Ioannidis et al. [40] to reduce unnecessary waste in research. Transitioning to electronic patient records and virtual assessments were also highly rated, supported by existing evidence of their efficacy in reducing emissions associated with travel and physical documentation [41,42,43]. However, refinement of database design, a measure with substantial potential to reduce data waste [9,44], was rarely prioritized by respondents. This suggests a possible underestimation of data inefficiencies and further illustrates the knowledge gap around less visible sources of carbon emissions. Other innovations, such as adjusting freezer storage temperatures, were also recognized, echoing the work of MGL and the Laboratory Efficiency Assessment Framework (LEAF) in reducing laboratory emissions [45]. While resource constraints and lack of organizational drive were cited as common barriers, these did not appear to significantly diminish willingness to engage with sustainability initiatives, which remained high (86%).

Many respondents highlighted the pivotal role of regulators in driving sustainability through clearer guidance and incentive structures. Suggestions included the removal of paper record requirements, minimization of kit sizes, increased permission for multi-use equipment, and reductions in packaging, all areas where study sponsors and industry stakeholders could exert influence. Furthermore, respondents strongly supported the integration of sustainability into funding criteria. Almost half agreed that mandatory inclusion of carbon footprint considerations in grant applications would incentivize more sustainable practices. These findings support calls by Adshead et al. [46] for funders to require researchers to justify carbon usage and support the broader view that systemic levers, rather than voluntary change alone, are needed to drive behavioral shifts within the research community.

A key strength of this study is its national scope and the inclusion of diverse professional roles, from clinicians to CRO staff. The mixed-methods approach enabled both quantitative insight and rich contextual data. However, the modest response rate (20.6%) introduces possible response bias; individuals more interested in sustainability may have been more likely to respond. Underrepresentation of surgeons and some trial support roles, despite their relevance to emissions-heavy activities, limits generalizability. The study also focused on self-reported perceptions rather than direct emissions data. Future research should include carbon footprint measurement across trial phases.

The findings of this study highlight clear, actionable opportunities for improving the environmental sustainability of cancer clinical trials in Ireland and internationally. Trial sponsors and coordinating bodies such as CTI should consider embedding sustainability requirements into protocol development, ethics review, and trial governance. Practical measures could include offering CPD-accredited training, enabling digital trial infrastructure, and reducing unnecessary travel and resource use. Funders and regulators also have a critical role to play in incentivizing greener practices through grant criteria and flexible regulatory guidance. By adopting a coordinated approach, Ireland can enhance research efficiency while aligning cancer trial conduct with national and EU climate goals.

## 5. Conclusions

This study provides the first national insight into sustainability awareness and attitudes in Irish cancer clinical trials. While formal training and awareness of carbon footprinting tools remain low, there is clear enthusiasm among professionals to adopt sustainable practices. Barriers such as unclear regulatory expectations, lack of institutional support, and perceived misalignment with trial compliance norms suggest the need for coordinated, system-wide interventions.

Ireland is well positioned to lead by example, showing that clinical research excellence and environmental responsibility are not mutually exclusive. CTI can play a pivotal role in catalyzing this shift through national leadership and collaboration, helping embed low-carbon principles into the research ecosystem. Advancing this agenda will depend on sustained engagement across clinical, academic, and policy sectors. It aligns directly with Ireland’s Climate Action Plan and the EU Green Deal, reinforcing the need for sustainable transformation across all aspects of healthcare, including research.

## Figures and Tables

**Table 1:** Survey Questions and Topics.

Question Number	Question Asked
1.	What is your main specialty or role within CTI?
2.	What is your current role in cancer research?
3.	Have you ever held the role of National Lead or Chief Investigator?
4.	In which province do you currently work?
5.	What is your current affiliation as a CTI stakeholder?
6.	Are you aware of any approaches or tools for calculating the carbon footprint of clinical trials (e.g., SCTG guidelines, NIHR calculator, My Green Lab certification)?
7.	To what extent do you believe that industry-sponsored trials consider carbon footprint during design?
8.	To what extent do you believe that academic trials consider carbon footprint during design?
9.	Please rank the following trial-related activities based on their perceived impact on a trial’s carbon footprint.
10.	Have you received any formal education or training on reducing the carbon footprint of clinical trials?
11.	How confident do you feel in your ability to advise on or implement sustainability measures in clinical trials?
12.	Would you be willing to participate in sustainability initiatives within clinical trials?
13.	Which of the following innovative measures do you think would have the greatest impact on reducing carbon emissions in trials?
14.	Do you have any additional practical suggestions for reducing a trial’s carbon footprint?
15.	Have you observed or been involved in any changes that have reduced the carbon footprint of a clinical trial?
16.	What do you think are the three main barriers to adopting sustainability measures in cancer clinical trials?
17.	Have you personally encountered any of the barriers you identified above? Please describe.
18.	What would have helped to overcome those barriers?
19.	What do you believe are the three best incentives for encouraging sustainability in clinical trials?
20.	Do you have any suggestions for accelerating the introduction of sustainable practices in cancer clinical trials?
21.	Do you have any suggestions for promoting or informing change regarding sustainability in cancer research?

**Table 2:** Professional penetration ratios of respondent to professional population.

Specialty/Role	Number Currently Working in the Cancer Clinical Trials Community	Number Who Participated in the Survey	Representation
Consultant Medical Oncologist	83	31	37%
Consultant Radiation Oncologist	51	14	27%
Consultant Surgeon	53	4	8%
Admin	figures not up to date	13	n/a
Data Manager/Bio Statistician	39	11	28%
Hematologist	54	9	17%
Translational Scientist	19	9	47%
Research Specialist	199	8	4%
Patient Consultant Committee member	13	4	31%

## Data Availability

Data are available upon reasonable request.
